# Monoclinic Silver Vanadate (Ag_0.33_V_2_O_5_) as a High‐Capacity Stable Cathode Material for Aqueous Manganese Batteries

**DOI:** 10.1002/advs.202406642

**Published:** 2024-08-13

**Authors:** Hyeonjun Lee, Hyungjin Lee, Jangwook Pyun, Seung‐Tae Hong, Munseok S. Chae

**Affiliations:** ^1^ Department of Nanotechnology Engineering Pukyong National University Busan 48547 Republic of Korea; ^2^ Department of Energy Science and Engineering DGIST Daegu 42988 Republic of Korea; ^3^ Department of Chemistry and Chemical Biology University of New Mexico New Mexico 87131 United States

**Keywords:** aqueous electrolytes, cathode materials, manganese batteries, silver vanadate

## Abstract

Aqueous rechargeable metal batteries have recently garnered considerable attention owing to their low cost, sufficient capacity, and the use of non‐flammable water‐based electrolytes. Among them, manganese batteries are particularly favored because of their stability, abundance, affordability, and high energy density. Despite their advantages, Mn storage host structures remain underexplored. Therefore, developing innovative host materials is crucial for advancing this field. In this paper, the study reports for the first time, the use of Ag_0.33_V_2_O_5_ as a cathode material in aqueous manganese batteries. The study explains the displacement/intercalation behavior of manganese and silver using electrochemical, structural, and spectroscopic analyses. Additionally, it is shown that cation (Ag^+^, Mn^2+^, H^+^) diffusion pathways can be simulated using diffusion‐barrier calculations. Finally, the study demonstrates high‐performance manganese batteries that exhibit a remarkable reversible capacity of ≈261.9 mAh g^−1^ at a current of 0.1 A g^−1^ and an excellent cycle retention of 69.1% after 2000 cycles at a current density of 1.5 A/g. The findings of this study contribute to the advancement of aqueous manganese battery technology, offering a promising pathway for developing safer, more cost‐effective, and high‐performance energy storage systems.

## Graphical abstract

The complex manganese (Mn) storage behavior of Ag_0.33_V_2_O_5_ is clarified using a methodology that combines electrochemical and structural characterization, elemental analysis, and migration barrier energy calculations for the diffusion of silver (Ag), Mn, and protons. While Ag precipitation on V_2_O_5_ can enhance the electrochemical activity, the generation of Mn(OH)_2_ can inhibit it.

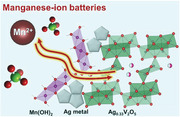

## 1 Introduction

Aqueous rechargeable batteries are gaining considerable attention as promising solutions to the safety problems associated with traditional batteries owing to their high stability, affordability, and eco‐friendliness.^[^ ^1^ ^]^ Their use of water‐based electrolytes considerably reduces the risk of fire and explosion, rendering them an ideal choice for a broad array of applications ranging from portable electronics to large‐scale energy storage systems. Moreover, the inherent safety features and low environmental impact of these batteries are aligned with the increasing global focus on sustainable green energy technologies.^[^ ^2^ ^]^

Traditionally, aqueous rechargeable batteries have used iron,^[^ ^3^ ^]^ zinc,^[^ ^4^ ^]^ and aluminum metal anodes^[^ ^5^ ^]^ because of their stability and high theoretical specific capacities. However, these methods have limitations such as the lower operating voltage of iron and increased polarization owing to the passivation layer on the aluminum anode.^[^ ^6^ ^]^

Aqueous manganese (Mn) batteries (MBs) are promising alternatives because of their intrinsic characteristics. Theoretically, Mn offers several advantages, including its gravimetric and volumetric capacity (976 mAh g^−1^ and 7250 mAh cm^−3^), abundance, affordability, and low redox potential (−1.18 V vs SHE).^[^ ^7^ ^]^ Moreover, they can be safely assembled in air, which improves the overall process efficiency. Despite these advantages, Mn storage host structures have not been extensively reported due to their relatively recent development, and current reports indicate that their performance is not outstanding. Due to this reason, only a few intercalation‐based electrode materials have been reported such as V_2_O_5_,^[^ ^8^ ^]^ Mn_0.18_V_2_O_5_·nH_2_O,^[^ ^9^ ^]^ Al_0.1_V_2_O_5_·1.5H_2_O,^[^ ^10^ ^]^ nickel hexacyanoferrate,^[^ ^8^ ^]^ and Mo_6_S_8._^[^ ^8^ ^]^ Consequently, it is essential to develop innovative new host materials.

Vanadium (V)‐based host materials are widely used as transition metals in multivalent‐ion batteries because of their broad redox ranges.^[^ ^11^ ^]^ In particular, hydrated layered V_2_O_5_ is commonly used, although it can suffer from poor thermal stability and degrade battery performance rapidly when water is lost from its structure.^[^ ^12^ ^]^ Consequently, research on V‐based materials without crystal water is essential to improve their thermal and cycle stabilities compared with existing materials.

**Figure 1:**
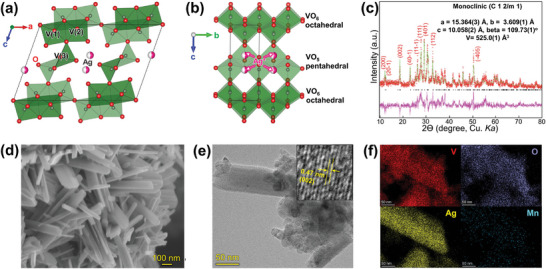
Characterization of Ag_0.33_V_2_O_5_ cathode: Crystal structure of AgVO along the a) a‐c‐plane, b) b‐c‐plane. c) XRD Rietveld refinement results. d) SEM image of AgVO nanobelt. e) TEM image and high‐resolution lattice fringe (inset). f) TEM‐EDX elemental mapping for V (red), O (purple), Ag (yellow), and Mn (cyan).

Ag_0.33_V_2_O_5_ (AgVO) is a mixed metal oxide composed of silver (Ag), vanadium (V), and oxygen (O) with a three‐dimensionally bonded structure,^[^ ^13^ ^]^ which is characterized by the storage of cations in a one‐dimensional (1D) tunnel array based on three V sites and one Ag site (Table S1, Supporting Information), as depicted in **Figure** **1**a,b. The structural configuration exhibits two distinct forms—that is, one featuring a linear chain of VO_6_ octahedra and the other featuring a composition of VO_5_ square pyramids. The linear chains form a zigzag double‐chain configuration, sharing edges and aligning parallel to the b‐axis, which maintains stable connectivity. Within the unit cell, two identical locations are created at ≈2 Å intervals on the Ag site. However, these equivalent locations cannot be occupied simultaneously, thereby limiting their maximum occupancies by half. Owing to its structural advantages, AgVO has already been used in various ion‐battery systems, including lithium,^[^ ^14^ ^]^ calcium,^[^ ^15^ ^]^ magnesium,^[^ ^16^ ^]^ and zinc.^[^ ^13^, ^17^ ^]^ The ionic radius of silver, ≈1.1 Å, is incorporated into the vanadium oxide structure. When the silver is removed, a wide cavity suitable for accommodating various cations is formed. Additionally, silver precipitates on the surface, reducing electrical resistance. Comparing energy density per unit volume, silver vanadate is a heavy electrode material for battery systems.

In this study, we demonstrated that AgVO has considerable potential as a high‐performance cathode material for aqueous MBs. The displacement/intercalation behavior of Mn and Ag was elucidated through a combination of electrochemical characterization, structural analyses, spectroscopic analyses, and migration barrier calculation techniques. Consequently, AgVO exhibited high Mn storage performance with a reversible capacity of ≈261.9 mAh g^−1^ at a current of 0.1 A g^−1^, along with excellent cycle retention. Our findings of this study contribute to the advancement of aqueous manganese battery technology, offering a promising pathway for developing safer, more cost‐effective, and high‐performance energy storage systems.

## 2 Results and Discussion

### 2.1 Ag_0.33_V_2_O_5_ Synthesis and Characterization

To obtain high‐purity AgVO, solid‐state synthesis was conducted at 400 °C for 1 h under optimal conditions. Synthesis at a higher temperature (500 °C) led to an increase in particle size, potentially decreasing electrochemical reactivity, while synthesis at a lower temperature (300 °C) resulted in reduced crystallinity (Figures S1 and S2, Supporting Information). The detailed synthesis procedures for AgVO are provided in the Supporting Information. The as‐synthesized powder was analyzed using X‐ray diffraction (XRD), scanning electron microscopy (SEM), and transmission electron microscopy (TEM) for characterization purposes. The analysis of the powder's XRD pattern (Figure 1c) verified the creation of a monoclinic phase belonging to the C2/m space group (a = 15.371(5) Å, b = 3.6086(7) Å, c = 10.047(2) Å and β = 109.74°(2)). These results are consistent with those of previous studies,^[^ ^13b^ ^]^ detailed crystallographic information of which is provided in Table S1 (Supporting Information).

The nanobelt morphology of AgVO (of thickness ≈100 nm), was revealed through SEM and TEM (Figure 1d,e). The high‐resolution TEM image (Figure 1e inset) displayed lattice fringes that matched the d(002) spacing of ≈0.47 nm. For the elemental quantitative and qualitative analyses, TEM mapping analysis using energy‐dispersive X‐ray spectroscopy (EDX) and SEM‐EDX spectra were used. Elemental mapping revealed that Ag, V, and O were uniformly distributed in the synthesized powder (Figure 1f). Small scattered manganese signals are also shown, as commonly occurs (sky blue). Also manganese cannot observed in SEM‐EDX spectra (Figure S3, Supporting Information). SEM‐EDX spectra showed that AgVO had 0.33 m Ag in the V_2_O_5_ structure (Figure S3, Supporting Information).

### 2.2 Electrochemical Manganese (Mn) Storage Performance

**Figure 2:**
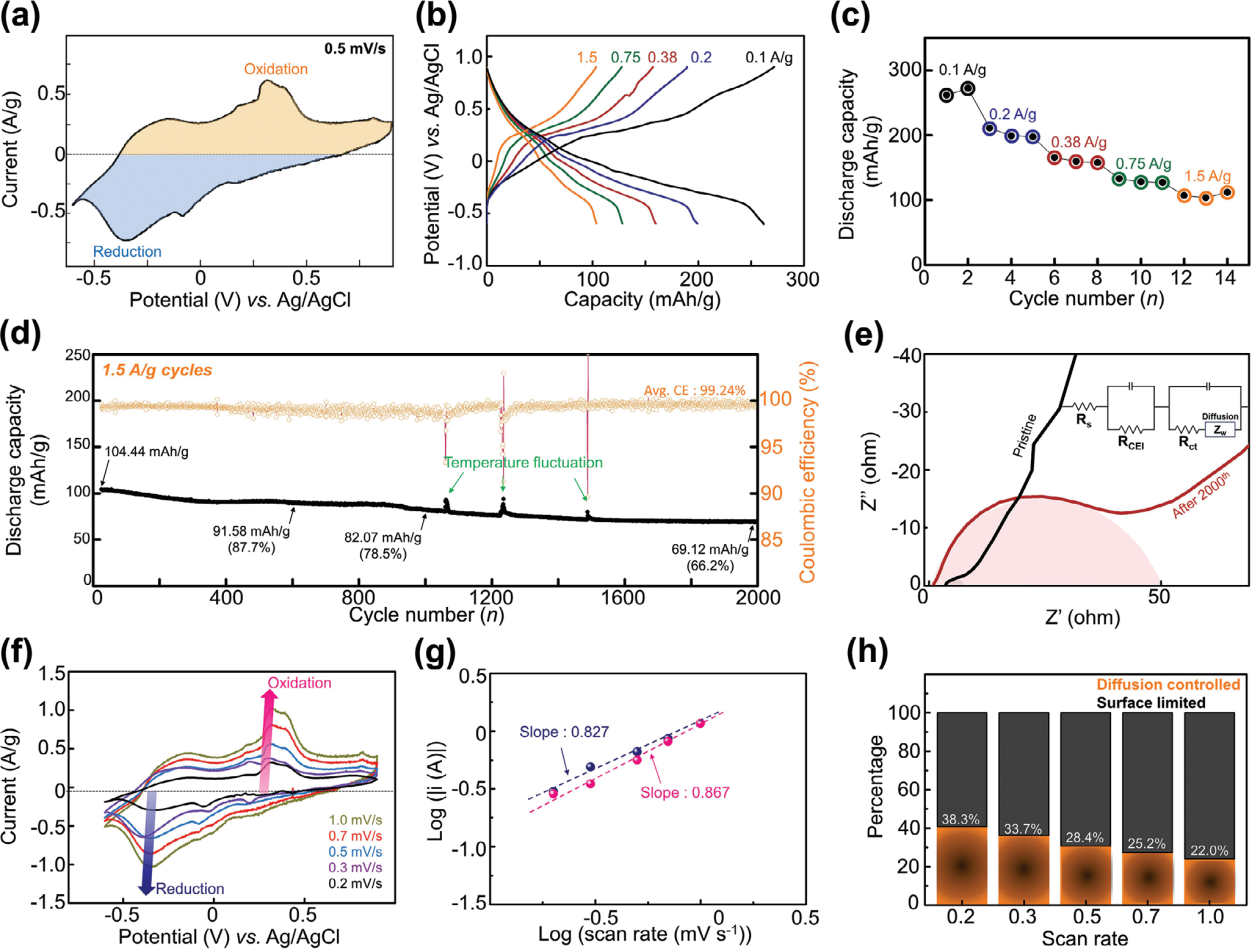
Electrochemical Mn storage performance of Ag_0.33_V_2_O_5_: a) CV curve at a scan rate of 0.5 mV s^−1^. b) GCD curves at different current densities. c) Rate performance at different current densities. d) Long‐term cyclability at 1.5 A g^−1^. e) Impedance spectra before and after 2000 cycles. (f) CV curves at scan rates in the range 0.2–1.0 mV s^−1^. g) Determination of *b*‐values from the relationship between the specific cathodic peak current and scan rate. h) Calculated ratio of diffusion‐controlled (orange) and surface‐limited (black) reactions for various scan rates.

Three‐electrode beaker‐type cells were used for all the electrochemical tests. The cyclic voltammetry (CV) curve, obtained at a scan rate of 0.5 mV s^−1^, demonstrated one major redox peak with several minor peaks (**Figure** **2**a). Specifically, three reduction peaks are observable at 0.18, −0.09, and −0.35 V (vs Ag/AgCl). The five oxidation peaks were observed at −0.2, 0.18, 0.31, 0.40, and 0.81 V (vs Ag/AgCl). According to the reaction mechanisms described in the elemental analysis section (vide infra), the complex peaks are attributed to a mixture of proton intercalation, manganese adsorption, and Mn(OH)_2_ formation reactions. In addition, the CV was stable, indicating that the redox reaction occurs without structural degradation (Figure S4, Supporting Information). At different current densities, the galvanostatic charge–discharge (GCD) curves exhibited a gradual variation (Figure 2b). Both the CV curves and galvanostatic profiles demonstrated excellent reversibility upon reaching the maximum current and capacity. Moreover, AgVO exhibited high‐rate capabilities, with specific capacities of 261.9, 199.4, 159.8, 128.3, and 103.8 mAh/g at 0.1, 0.2, 0.38, 0.75, and 1.5 A g^−1^, respectively (Figure 2c). To cross‐check the rate performance, we used the same cell (Figure S6, Supporting Information). When compared to other reported materials, AgVO exhibits a relatively high reversible capacity. For instance, it surpasses other cathodes such as Mo_6_S_8_ (≈90 mAh g^−1^ @ 0.5 A g^−1^),^[^ ^8^ ^]^ nickel hexacyanoferrate (≈43 mAh g^−1^ @ 0.5 A g^−1^),^[^ ^8^ ^]^ V_2_O_5_ (≈72 mAh g^−1^ @ 1 A g^−1^),^[^ ^8^ ^]^ and Mn_0.18_V_2_O_5_·nH_2_O (≈107 mAh g^−1^ @ 1 A g^−1^).^[^ ^9^ ^]^ However, our capacity and rate capabilities are lower when compared to the Al_0.1_V_2_O_5_·1.5H_2_O cathode.^[^ ^10^ ^]^ As shown in Figure 2c, it operated with similar electrochemical performance (capacity). The difference is that after the c‐rate test, another cycle at 0.1 A g^−1^ was performed. The capacity retention was 92.7% after 17 cycles even low current of 0.1 A g^−1^.

The discharge capacity during long‐term cycling was measured at a high current density of 1.5 A g^−1^ (Figure 2d). The initial capacity was 104.44 mAh g^−1^ and 66.2% (69.12 mAh g^−1^) after 2000 cycles, reflecting a highly stable cycle retention (Figure S5, Supporting Information). During the 2000 cycles, a high average Coulombic efficiency of 99.24% was observed. The remaining 0.76% might be attributed to water electrolysis from the electrolytes. Figure 2e shows the impedance plots before and after 2000 cycles. The cell internal resistance (R_s_) slightly decreased from 4.3 to 1.8 Ω, indicating that side reactions during cycling do not affect the interaction between our titanium current collector and electrode materials. Precipitated silver metal could potentially lower the resistance. These results suggest that the Ti current collector is stable in Mn(ClO_4_)_2_‐based aqueous electrolyte. For the pristine electrode, the charge‐transfer resistance (R_CEI_+R_ct_) was 8.3 Ω. However, after the activation process (2000 cycles), the resistance increased considerably (50.1 Ω). This might be due to some side products being generated and accumulating on the cathode material's surface (regarding the side reactions discussed later). Moreover, the Warburg slope decreased, indicating that the diffusion kinetics degraded during cycling, possibly due to the degradation of the cathode material.

To investigate the Mn intercalation reaction during the discharge–charge process, CV tests on the AgVO electrodes were conducted at scan rates of 0.2–1.0 mV s^−1^, the results of which are shown in Figure 2f. To explore the cation storage mechanism, the power law was employed as an analytical framework to distinguish between two predominant reaction paradigms^[^ ^18^ ^]^—that is, those governed by diffusion and those limited by surface interactions. The power‐law coefficient (b), which distinguishes between the capacitive behavior and diffusion‐controlled kinetics, was determined to be 0.808 for the reduction processes and 0.864 for the oxidation processes (Figure 2g; Figure S7, Supporting Information). This numerical evidence suggests that the storage capacity of AgVO is concurrently modulated by capacitive and diffusion‐driven reactions. Analysis revealed that ≈40.8% of the Mn ions underwent intercalation into the host matrix, whereas the remaining 59.2% exhibited characteristics indicative of the surface‐limited phenomena, attributed largely to the modest involvement of the electrostatic double layer (Figure 2h).^[^ ^19^ ^]^ Concurrently, an increase in the scan rate was shown to precipitate a notable increase in the surface capacitance, a phenomenon graphically illustrated in Figure S7 (Supporting Information).

### 2.3 Elemental Analyses During Manganese (Mn) Storage

**Figure 3:**
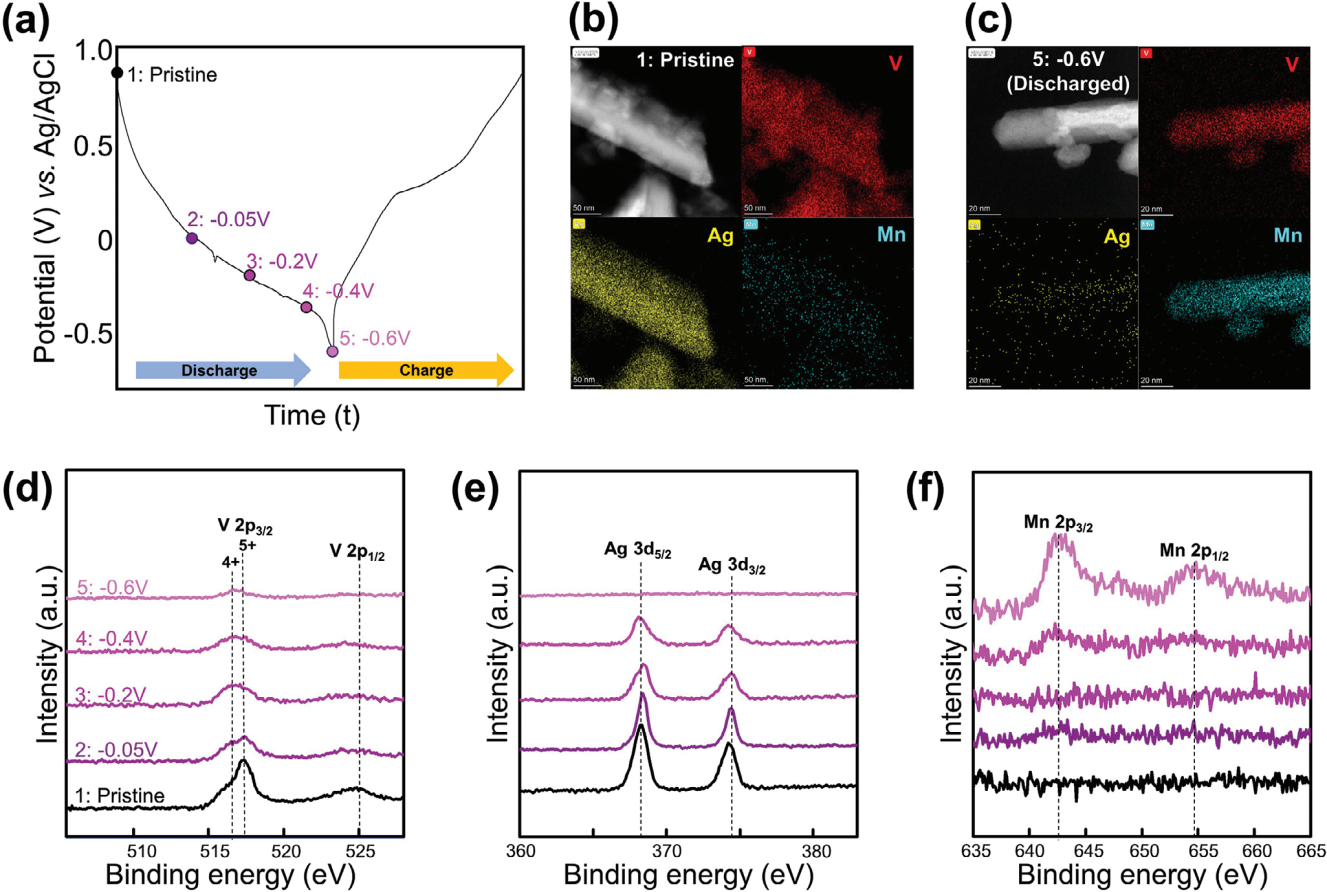
a) GCD curve from pristine to −0.6 V (vs Ag/AgCl) reference (measured point), TEM‐EDX elemental mapping of b) pristine and c) fully discharged Ag_0.33_V_2_O_5_. XPS spectra of d) V 2p, e) Ag 3d, and f) Mn 2p.

Elemental analysis during the discharge–charge process was performed using TEM (Themis Z) with EDX and X‐ray photoelectron spectroscopy (XPS, ESCALAB 250Xi), as shown in **Figure** **3**. The galvanostatic discharge–charge profiles of the samples are shown in Figure 3a. Figure 3b,c show the pristine (point 1) and discharged (point 5, −0.6 V vs Ag/AgCl) states of the electrode, respectively. Mn, initially absent in the pristine state, is uniformly distributed in the discharged samples, suggesting that the redox reaction may involve the intercalation of Mn ions. However, the Ag disappears after discharge.

The XPS profiles obtained during the discharge process of the AgVO electrodes at five points (0.8, −0.05, −0.2, −0.4, and −0.6 V vs Ag/AgCl) are shown in Figure 3d–f. These profiles provide crucial insights into the oxidation states of V, Ag, and Mn. Active V redox couples and increasing Mn peaks are evident in the spectra (Figure 3d,f). Within the 505–528 eV range, the V 2p spin‐orbitals of the raw electrode show three distinct peaks at 517.4 (V^5+^ 2p_3/2_), 515.6 (V^4+^ 2p_3/2_), and 525.0 eV (V^5+^ 2p_1/2_), aligning with previous findings on other cations. Additionally, the intensity of the Ag peaks decreases, suggesting that Ag from the AgVO structure may dissolve in the electrolyte or become difficult to detect because of the formation of surface products during the discharge process (Figure 3e). After the charge process, the XRD data shows partially return to the pristine state, although slight Mn(OH)_2_ peaks still remain (Figure S8, Supporting Information). The precipitated silver metals do not work reversibly. Regarding the XPS analysis of the charged electrode, the oxidation state of vanadium returns to 5+, and the manganese peaks decrease but are not completely removed. This indicates that the Mn(OH)_2_ reaction is partially reversible during the redox process (Figure S9, Supporting Information).

However, the V and Ag peaks decrease simultaneously, indicating that the reduction in the V and Ag peaks in the XPS analysis is a result of the generation of the coating layer of the Mn complex on the surface. Consequently, bulk analysis using XRD was conducted, which confirmed the formation of Ag metal, maintenance of the anode structure, and generation of the side reactions.

**Figure 4:**
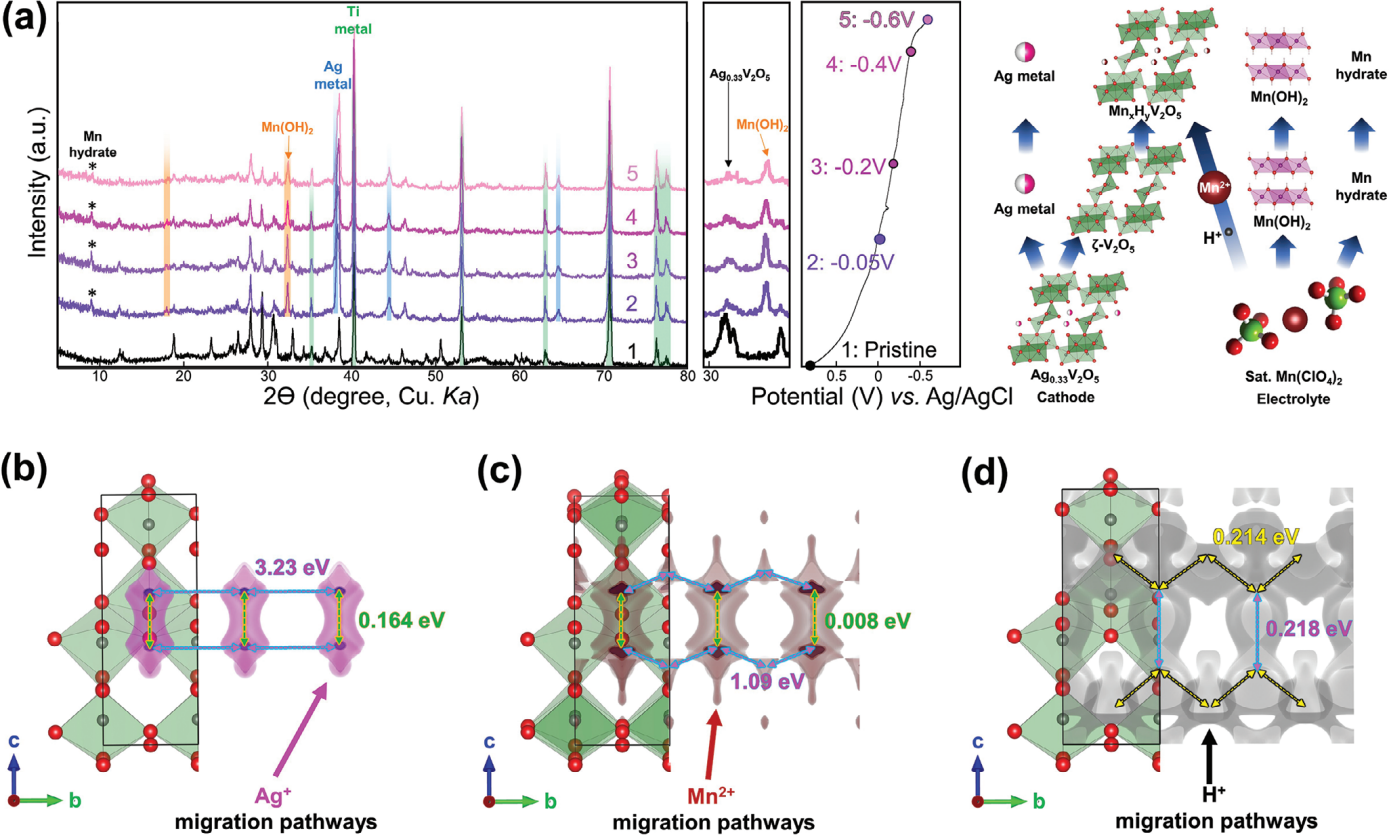
a) XRD profile during the discharge process, along with the corresponding electrochemical profile and their reaction mechanism. Calculated migration barriers and paths for b) Ag, c) Mn, and d) proton ions in the b‐c‐plane.

To clarify the reaction mechanism, the electrochemical charge storage mechanism of the AgVO structure (which combines the displacement and intercalation processes), was investigated using the powder XRD data (**Figure** **4**a). The pristine state shows only Ag_0.33_V_2_O_5_ and titanium current collector peaks. However, during the discharge process, new peaks appear, which can be explained by the following three reaction mechanisms:

1. Around 9° (2θ), which is typically associated with Mn‐hydrated byproducts, a similar phenomenon is evident in aqueous zinc‐ion battery systems;^[^ ^20^ ^]^
2. Mn(OH)_2_ peak generation during discharge (which provides strong evidence of proton insertion into the structure). The electrolyte reaction equations can be expressed as follows:
(1) $$\begin{array}{ccc} & & 2Mn^{2+}\left(\mathrm{aq}\right)+4\mathrm{Cl}{O_{4}}^{-}\left(\mathrm{aq}\right)+2H^{+}\left(\mathrm{aq}\right)+2OH^{-}\left(\mathrm{aq}\right)\\ & & \to \mathrm{Mn}{\left(\mathrm{OH}\right)}_{2}\left(s\right)+2\mathrm{Cl}{O_{4}}^{-}\left(\mathrm{aq}\right)+Mn^{2+}\left(\mathrm{aq}\right)+2H^{+}\left(\mathrm{aq}\right)\;\;\\ & & \left(\mathrm{insertion}\;\mathrm{to}\;Ag_{0.33}V_{2}O_{5}\;\mathrm{host}\;\mathrm{structure}\right) \end{array}$$
3. Precipitation of Ag ions to form Ag metal, indicated by the generation of peaks ≈38° (2θ). This suggests a combination of displacement and intercalation mechanisms involving various cations. The cathodic reaction equations can be expressed as follows:
(2) $$\begin{array}{ccc} Ag_{0.33}V_{2}O_{5}\left(s\right) & & +xMn^{2+}\left(\mathrm{aq}\right)+yH^{+}\left(\mathrm{aq}\right)\\ & & \to Mn_{x}H_{y}V_{2}O_{5}\left(s\right)+\mathrm{Ag}\left(s\right) \end{array}$$

Based on XRD analyses, the decrease in Ag and V signals in the XPS can be attributed to surface coating by Mn(OH)_2_ and Mn byproducts. This observation is consistent with the decrease in the Ag_0.33_V_2_O_5_ peak intensity in the XRD pattern during the discharge process. Consequently, Ag_0.33_V_2_O_5_ exhibits co‐insertion of protons and Mn into the structure, with Ag from Ag_0.33_V_2_O_5_ precipitating onto the ζ‐V_2_O_5_ surface. Additionally, the cathodes are covered with Mn byproducts (such as Mn(OH)_2_ and Mn hydrates).

Ag migration barriers and pathways were calculated using the softBV‐GUI simulation tool^[^ ^21^ ^]^ (Figure 4b–d), with potential migration pathways depicted along the b‐c‐plane. Ag diffusion along the b‐axis was hindered by the high migration barrier (3.23 eV (Figure 4b)). Consequently, Ag ions could not readily diffuse along the b‐axis, primarily because of ion‐hopping reactions.

The Mn migration barriers were calculated using the same method (Figure 4c). Several possible migration pathways were identified for Mn—that is, one along the b‐axis, and the other along the b‐axis. The migration barriers for Mn were considerably lower than those for Ag, indicating the easier diffusion of Mn within the structure. However, with a migration barrier of 1.09 eV, Mn diffusion was still considerably high for diffusion reactions, suggesting that Mn may prefer surface redox reactions and ion hopping within the structure.

The diffusion barriers for protons are considerably lower than those for Mn and Ag ions, facilitating fast diffusion kinetics and enhancing the overall electrochemical performance of the AgVO structure (Figure 4d). Protons can diffuse along the 1D b‐axis (0.214 eV) and across the c‐axis (0.219 eV), benefiting from their lower migration barriers. Consequently, proton insertion is preferred over Mn insertion into the structure, leading to Mn side reactions, such as Mn(OH)_2_ generation.

**Figure 5:**
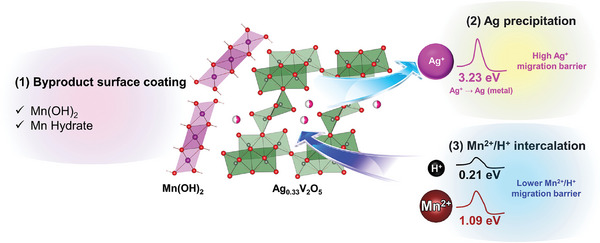
Schematic of byproduct generation on the cathode surface and the displacement/intercalation mechanism, Ag displacement, and Mn/proton insertion processes.

**Figure** **5** illustrates the suggested reaction mechanism during the initial discharge cycle. When Ag_0.33_V_2_O_5_ is discharged into the Mn electrolyte, the three reactions occur simultaneously. Mn byproducts are formed, which can be attributed to proton intercalation. These byproducts (Mn(OH)_2_ and hydrated Mn) cover the surface of the cathode material, making the precipitation of Ag less evident in various surface analyses. However, the Ag precipitation reaction is clearly visible in the XRD pattern.

TEM (Figure S10, Supporting Information) was used to capture the areas of the cathode material coated with these byproducts. Additionally, competitive redox reactions occurred between protons and Mn. Based on their migration energy, proton ions are more likely to be preferred over Mn ions. Owing to its higher migration energy, Mn diffuses via hopping reactions within the structure and primarily participates in surface redox reactions.

## 3 Conclusion

In this study, we introduced Ag_0.33_V_2_O_5_ as a promising cathode material with high capacity and stability for use in aqueous ion batteries. Through electrochemical characterization, structural analysis, and spectroscopic analysis, we examined the displacement/intercalation behavior of Mn and Ag. Additionally, we simulated the cation diffusion pathways using diffusion‐barrier calculation techniques. This material exhibited a first discharge capacity of 261.9 mAh g^−1^ at a current of 0.1 A g^−1^ and an average redox voltage of ≈1.3 V (vs Mn/Mn^2+^). Furthermore, it demonstrated excellent cycle retention, retaining 69.1% of its capacity over 2000 cycles at a current of 1.5 A g^−1^.

Structural and elemental analyses clarified the reaction mechanism, revealing a combination of the displacement and intercalation behaviors of Mn and Ag. Additionally, proton insertion could lead to Mn byproducts such as Mn(OH)_2_ and Mn hydrates, which suppressed electrochemical reaction activities. Consequently, proper electrolyte research is crucial to achieving high‐performance Mn‐ion batteries.^[^ ^7c^ ^]^

Our findings offer valuable insights into cathode research for Mn‐ion batteries, including the reaction mechanisms and byproduct formation. Additionally, this is the first report on the use of Ag_0.33_V_2_O_5_ as a cathode material for Mn‐ion batteries, which, to the best of our knowledge, has rarely been reported.

However, this is early‐stage information. For better Mn battery performance, various future research efforts are needed, such as electrolyte research and the modification of cathode materials through doping and carbon composites. For practical applications, the utilization of Mn metal should be considered in the future, making electrolyte research mandatory.

Overall, these findings could promote the design and development of next‐generation high‐energy and stable cathode materials for future battery systems.

## 4 Experimental Section

### Material Synthesis and Characterization of Silver Vanadate

Ag_0.33_V_2_O_5_ powder was synthesized using the sol‐gel method. 1 m V_2_O_5_ (≥99.6%, Sigma‐Aldrich), 0.33 m AgNO_3_ (≥99.9%, Alfa Aesar), and 4 m oxalic acid (≥99.5%, Alfa Aesar) were dissolved in 200 mL of deionized water, centrifuged at 200 rpm, and dried at 90 °C overnight. The prepared gel powder was baked at 400 °C for 1 h in air. The powder morphology was examined by SEM (Hitachi 8020) and TEM (FEI, Themis Z) equipped with EDX.

### Electrochemical Characterization

The cathode comprised Ag_0.33_V_2_O_5_ powder, Super C conductive carbon (Timcal), and poly(vinylidene fluoride) binder (Kureha Co.) at a ratio of 8:1:1 (by weight). These powders were dispersed in N‐methyl‐2‐pyrrolidone and coated onto 32 µm titanium foil (Sigma‐Aldrich). The coated electrodes were dried at 80 °C and pressed using an electrode pressing device. The loading mass of AgVO was ≈2 mg for each electrode, excluding the conductive carbon and binder. Activated carbon was used as the counter, and Ag/AgCl was used as the reference electrode, separated by a glass fiber separator paper (Whatman). A saturated Mn perchlorate solution (Sigma‐Aldrich) in deionized water was used as the aqueous electrolyte.

All electrochemical measurements (three‐electrode cell) were conducted using a beaker‐type cell with a Ti current collector and saturated Mn perchlorate aqueous solution as the electrolyte, along with an Ag/AgCl reference. Cyclic voltammograms, galvanostatic charge/discharge, and impedance spectra were measured using a Biologic VMP‐3e. The impedance was measured in PEIS (potential) mode over a frequency range of 0.1 Hz to 200 kHz.

### Diffusion‐Barrier Calculation

The cation migration barriers were calculated using the softBV‐GUI simulation tool.^[^ ^21^ ^]^ The Ag, Mn ion, and proton migration characteristics were assessed using the crystal structure derived from Rietveld analysis based on powder XRD data using the General Structure and Analysis System (GSAS) crystallographic package.^[^ ^22^ ^]^ 3D diffusion pathways were visualized using VESTA software (ver. 3).^[^ ^23^ ^]^

## Conflict of Interest

The authors declare no conflict of interest.

## Supporting information

Supporting Information

## Data Availability

The data that support the findings of this study are available from the corresponding author upon reasonable request.
