# Supplementary material for: Monoclinic Silver Vanadate (Ag0.33V2O5) as a High‐Capacity Stable Cathode Material for Aqueous Manganese Batteries
Source: Adv Sci (Weinh). 2024 Aug 13;11(39):2406642. doi: 10.1002/advs.202406642 (PMC11496989; doi:10.1002/advs.202406642)
Supplement: Supplementary file 1 — Supporting Information [file ADVS-11-2406642-s001.docx]

Supporting Information

Monoclinic Silver Vanadate (Ag_0.33_V_2_O_5_) as a High-Capacity Stable Cathode Material for Aqueous Manganese Batteries

Hyeonjun Lee, Hyungjin Lee, Jangwook Pyun, Seung-Tae Hong, and Munseok S. Chae*


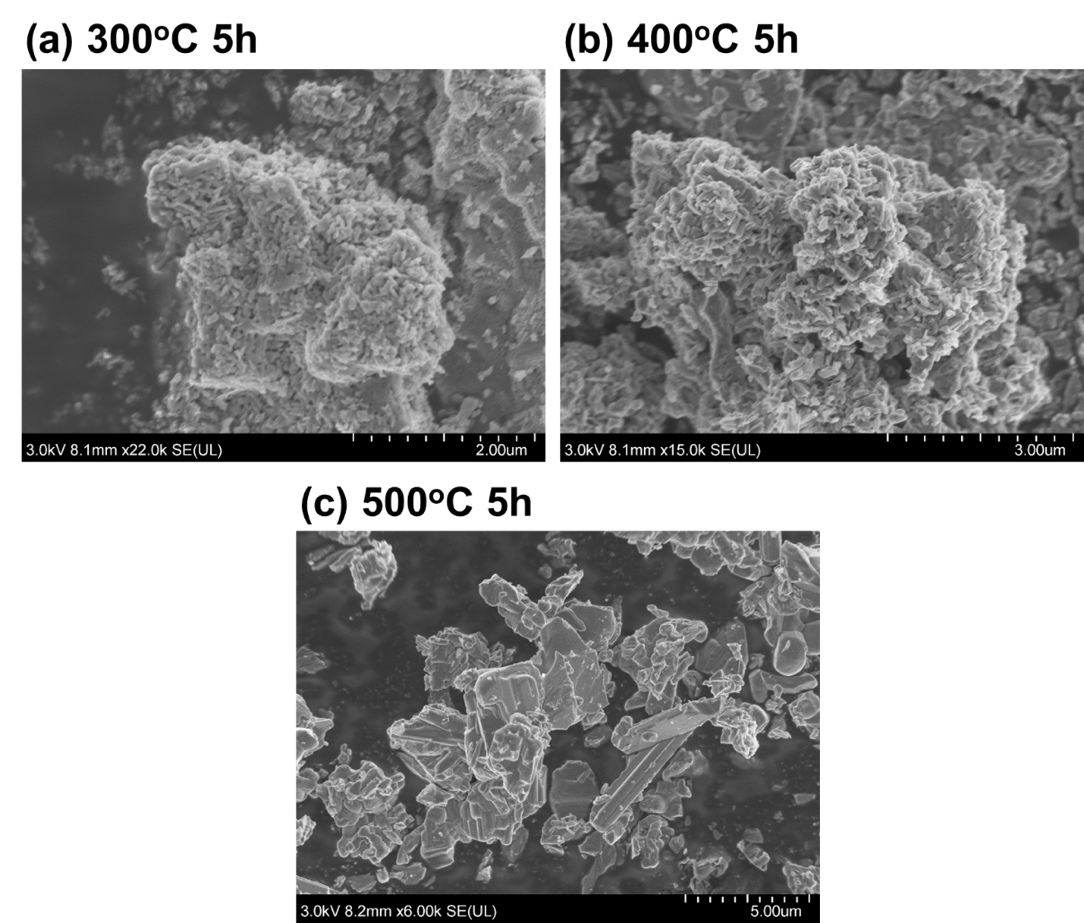
Figure S1. SEM images of Ag_0.33_V_2_O_5_ under various synthesis conditions at (a) 300 °C 5 h, (b) 400 °C 5 h, and (c) 500 °C 5 h.


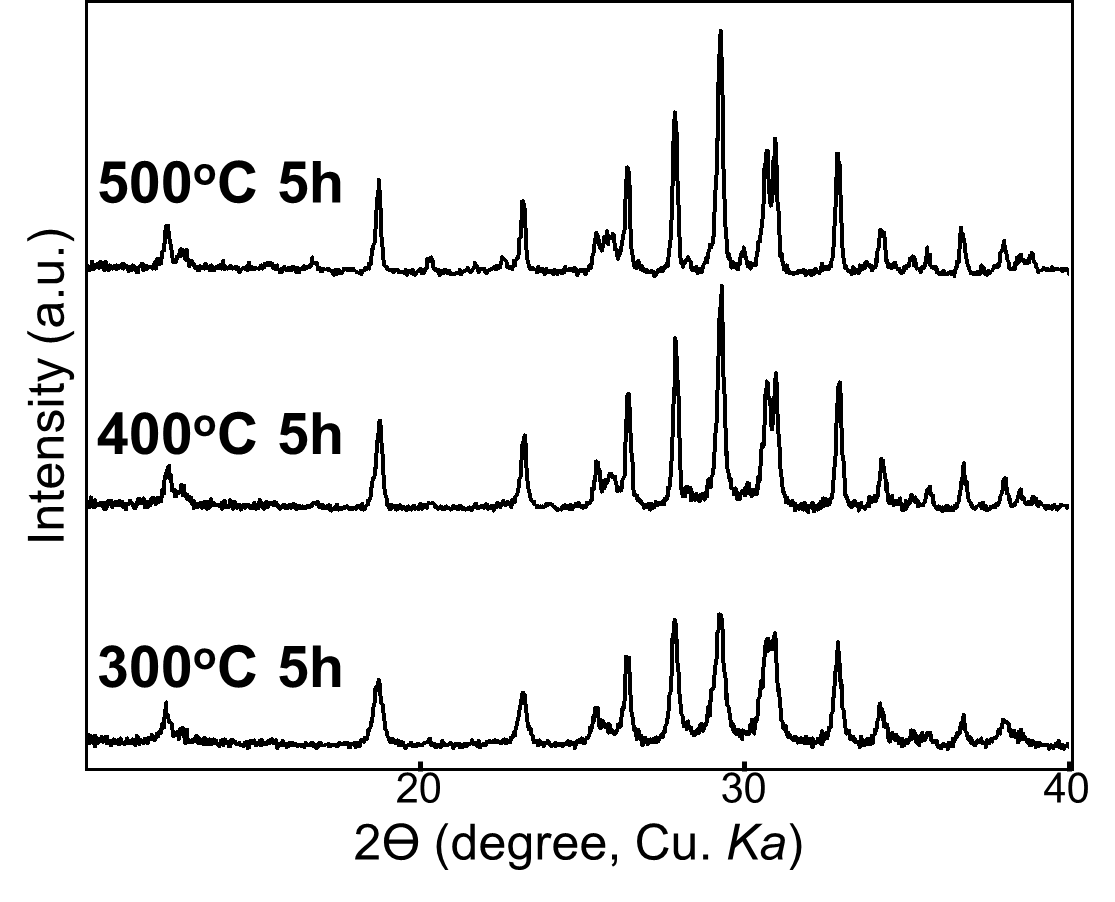


Figure S2. XRD profiles of Ag_0.33_V_2_O_5_ under various synthesis conditions 300 °C 5 h, 400 °C 5 h, and 500 °C 5 h.

**Table S1.** Crystallographic data and Rietveld refinement data for Ag_0.33_V_2_O_5_ by powder XRD data: atomic coordinates, site occupancies, and isotropic displacement parameters.

| Crystal System | | | | Monoclinic | | | |
| --- | --- | --- | --- | --- | --- | --- | --- |
| Space Group | | | | C 2/m (No 12) | | | |
| Lattice Parameter | | | | a = 15.371(5) Å, b = 3.6086(7) Å,  c = 10.047(2) Å, beta = 109.74(2)°_,_  V = 524.5(3) Å^3^ | | | |
| Atoms | x | y | Z | | Wyckoff | Occupancy | U_iso_ |
| Ag(1) | 0.989(1) | 0.0000 | 0.398(2) | | 4i | 0.500 | 0.050(3) |
| V(1) | 0.124(1) | 0.0000 | 0.142(2) | | 4i | 1.000 | 0.050(3) |
| V(2) | 0.344(1) | 0.0000 | 0.115(2) | | 4i | 1.000 | 0.050(3) |
| V(3) | 0.289(1) | 0.0000 | 0.404(2) | | 4i | 1.000 | 0.050(3) |
| O(1) | 0.000 | 0.0000 | 0.0000 | | 2a | 1.000 | 0.050(3) |
| O(2) | 0.106(5) | 0.0000 | 0.297(6) | | 4i | 1.000 | 0.050(3) |
| O(3) | 0.093(5) | 0.5000 | 0.110(4) | | 4i | 1.000 | 0.050(3) |
| O(4) | 0.267(5) | 0.0000 | 0.253(7) | | 4i | 1.000 | 0.050(3) |
| O(5) | 0.470(4) | 0.0000 | 0.211(6) | | 4i | 1.000 | 0.050(3) |
| O(6) | 0.331(3) | 0.5000 | 0.035(6) | | 4i | 1.000 | 0.050(3) |
| O(7) | 0.365(3) | 0.0000 | 0.510(6) | | 4i | 1.000 | 0.050(3) |
| O(8) | 0.275(4) | 0.5000 | 0.439(6) | | 4i | 1.000 | 0.050(3) |

* *R*_p_ = [18.1](1_publ%20_pd_proc_ls_prof_R_factor)%, *R*_wp_ = [23.7](1_publ%20_pd_proc_ls_prof_wR_factor)%, *R*_exp_ = [21.9](1_publ%20_pd_proc_ls_prof_wR_expected)%, *R*(*F*^2^) = [19.8%](1_publ%20_refine_ls_R_Fsqd_factor), χ^2^ = 1.19

**
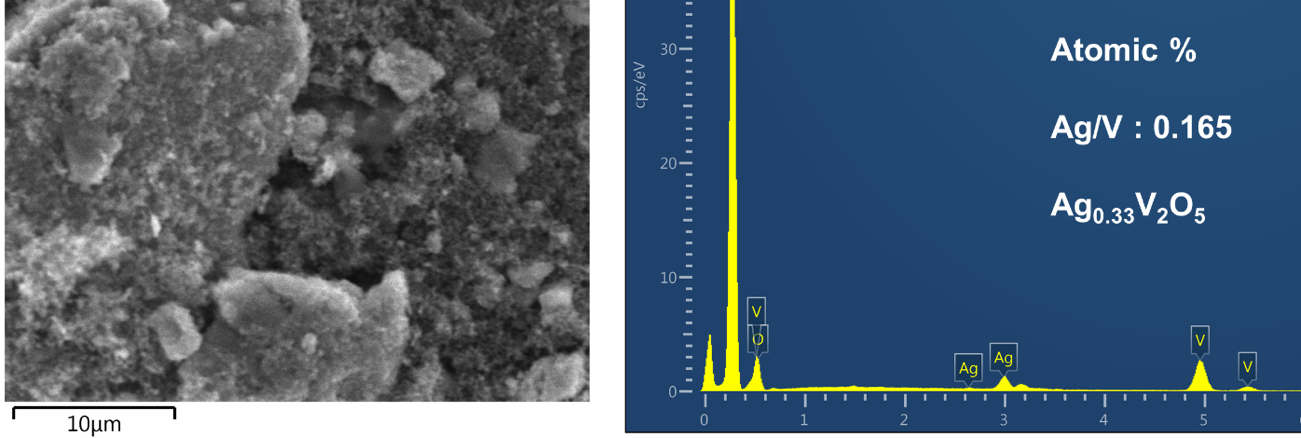
**

**Figure S3.** SEM-EDX spectra and quantification data of Ag_0.33_V_2_O_5_, synthesized at 400 °C 1 h.


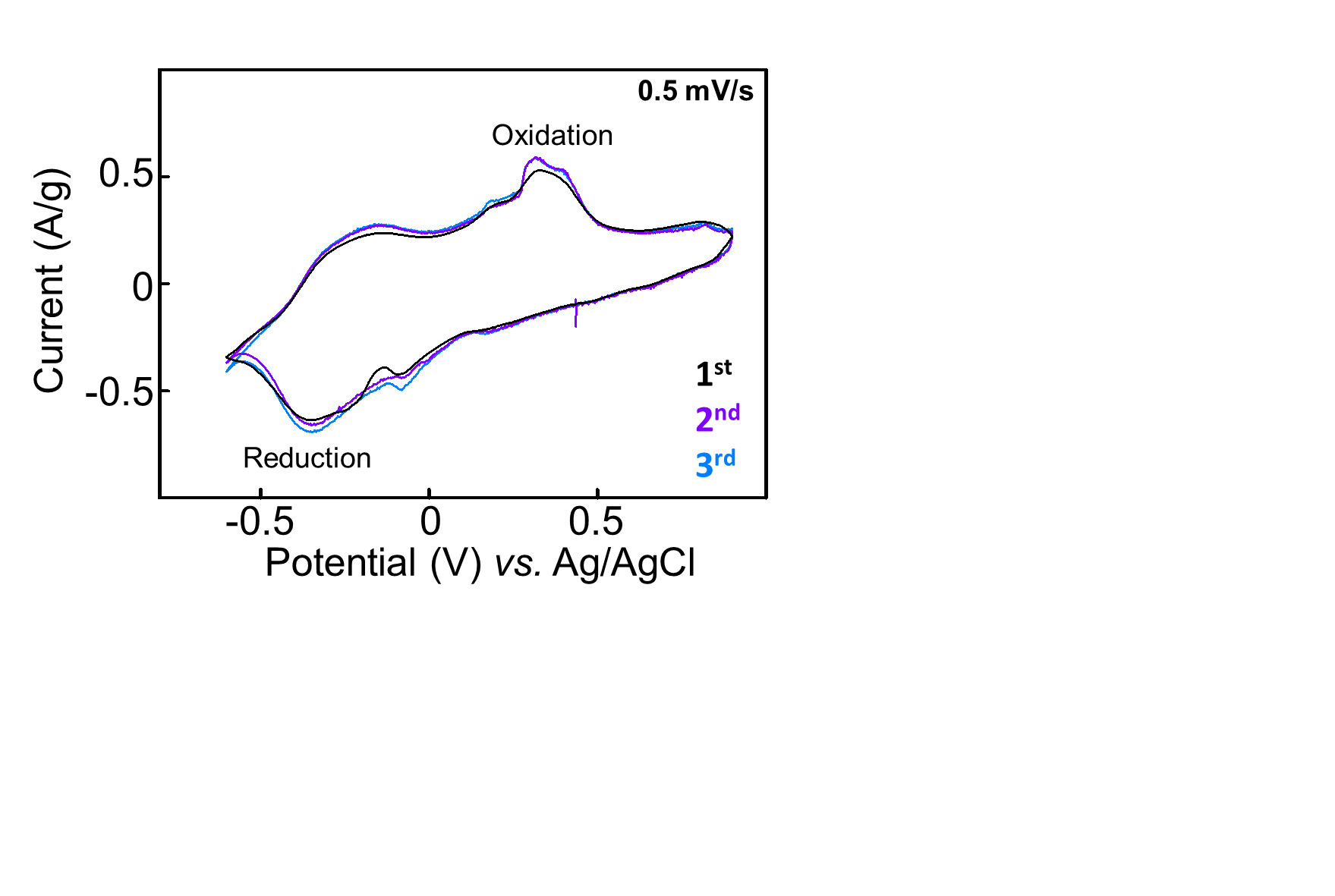


Figure S4. CV curves during three cycles at a scan rate of 0.5 mV/s.


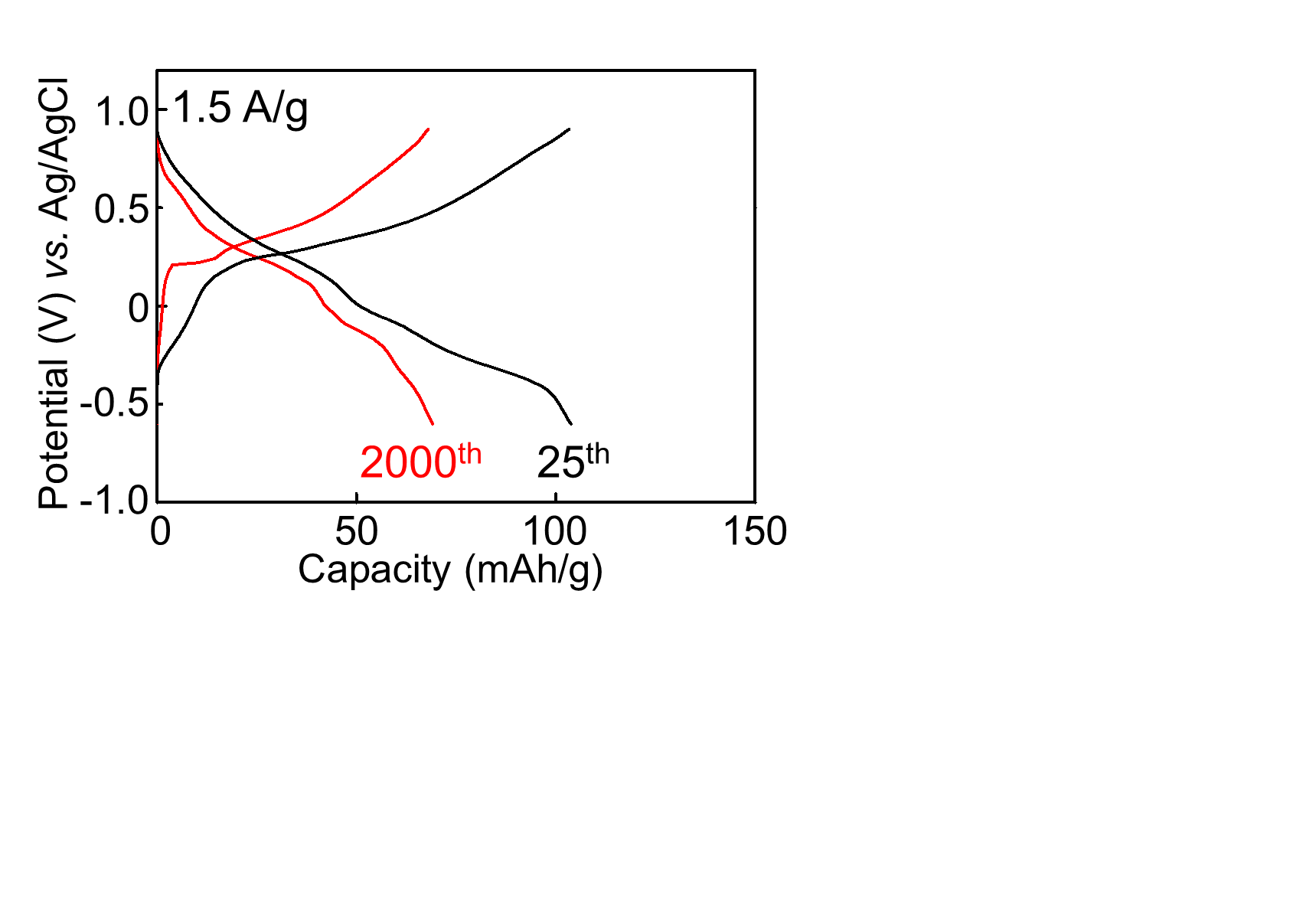
Figure S5. Galvanostatic discharge–charge profiles of the 25^th^ and 2000^th^ cycles.


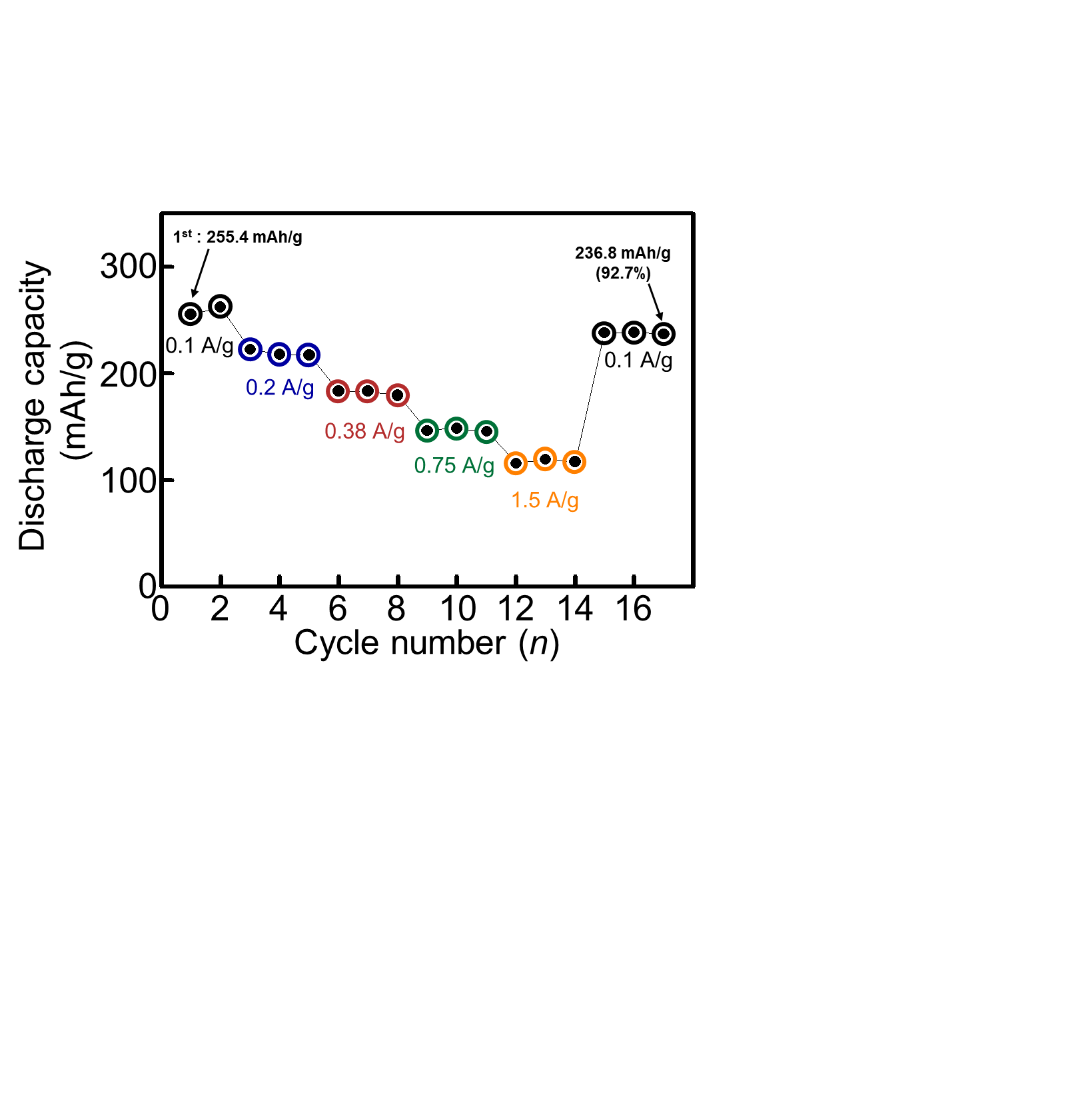


Figure S6. Rate performance at different current densities.

.


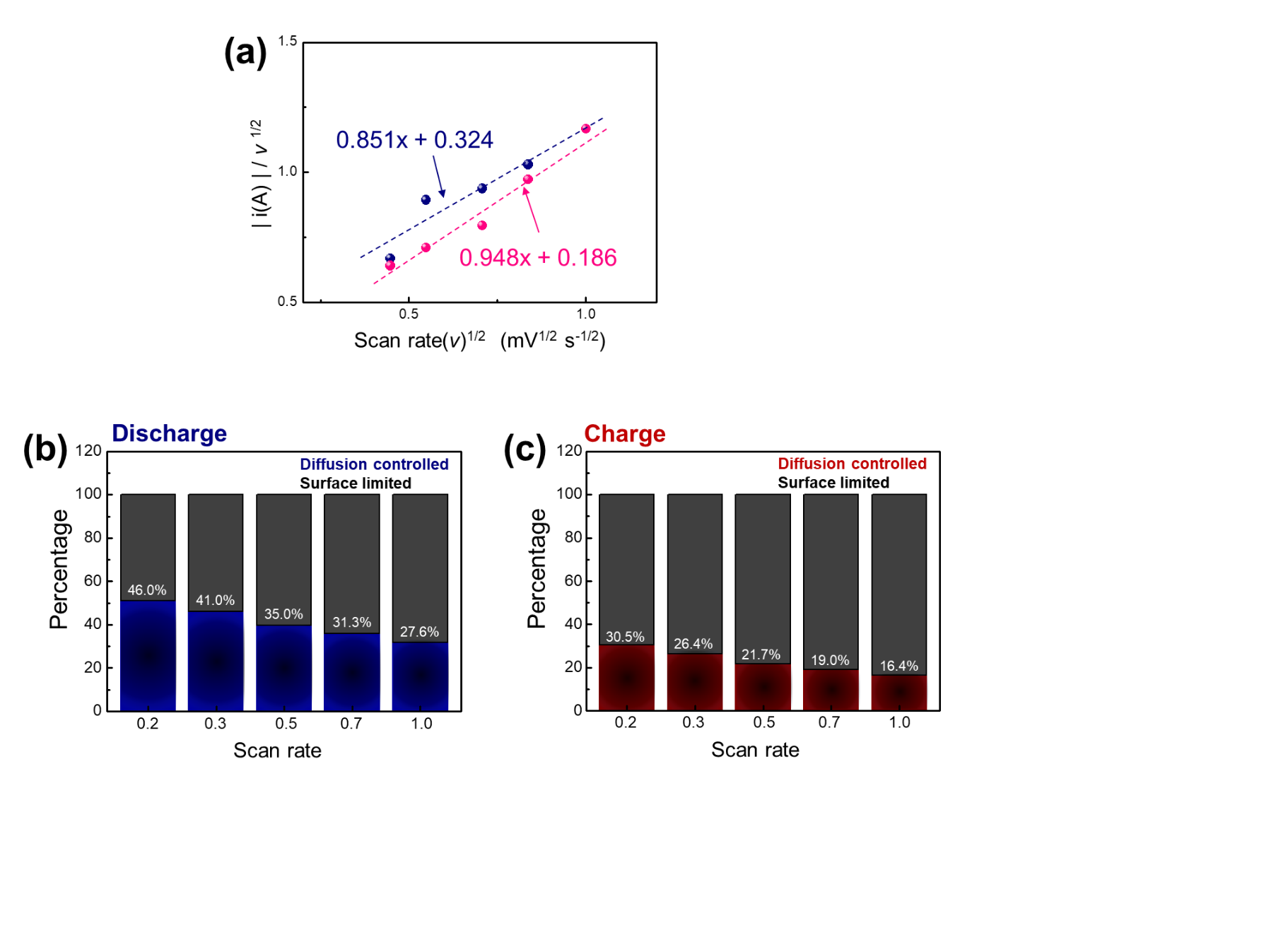


Figure S7. (a) Cathodic peak current dependence on the scan rate (obtained to determine the capacitive and intercalation contributions to energy storage). Calculated intercalation/adsorption ratios with various scan rates for (b) discharge and (c) charge processes.


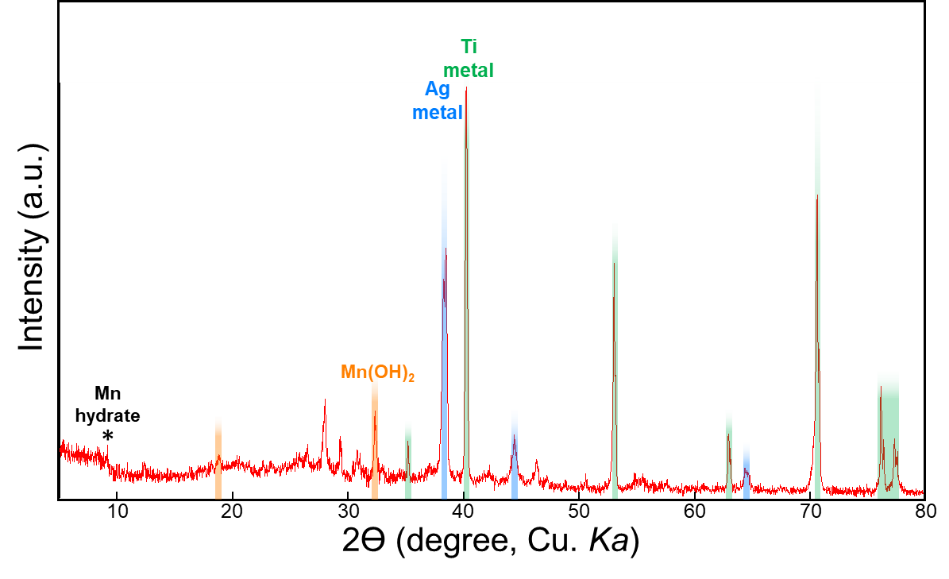


**Figure S8.** XRD profile of after charged electrode.

**
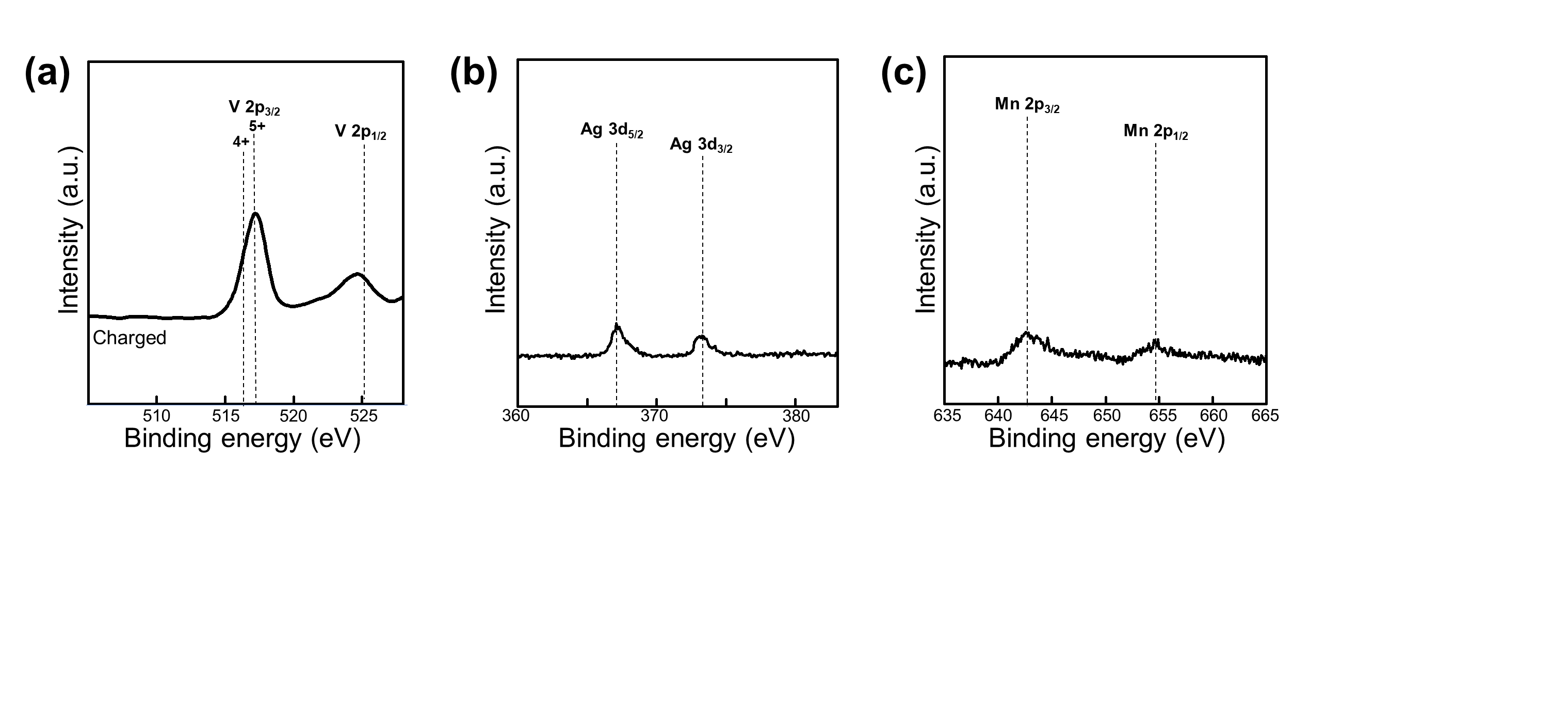
**

**Figure S9.** XPS spectra of after charged electrode : (a) V 2p, (b) Ag 3d, and (c) Mn 2p.

**
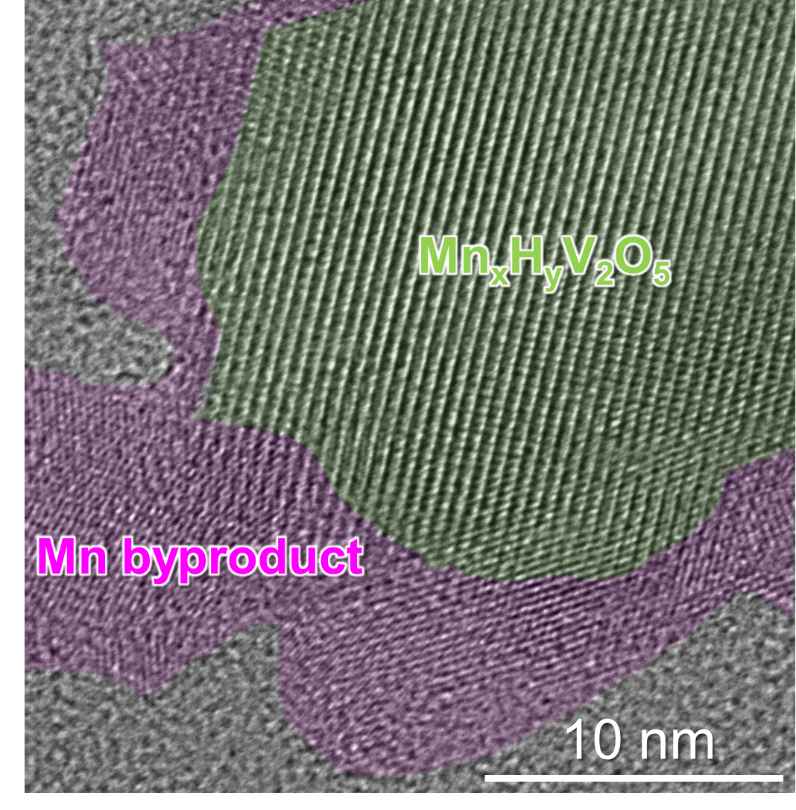
Figure S10.** Local high-resolution TEM image of the discharged sample.
